# Socio-economic burden and resource utilisation in Italian patients with chronic urticaria: 2-year data from the AWARE study

**DOI:** 10.1016/j.waojou.2020.100470

**Published:** 2020-12-08

**Authors:** Oliviero Rossi, Angelo Piccirillo, Enrico Iemoli, Annalisa Patrizi, Luca Stingeni, Stefano Calvieri, Massimo Gola, Paolo Dapavo, Antonio Cristaudo, Leonardo Zichichi, Laura Losappio, Fabiana Saccheri, Elide Anna Pastorello

**Affiliations:** a Azienda Ospedaliera Universitaria Careggi, SOD Immunoallergologia, Florence, Italy; b AO Ospedale San Carlo di Potenza, Italy; c ASST FBF Sacco, Milan, Italy; d UOC Dermatologia Metropolitana, DIMES, University Bologna, Italy; e Section of Dermatology, Department of Medicine, University of Perugia, Italy; f AOU Policlinico Umberto I, Rome, Italy; g AOU Ospedale Piero Palagi/IOT UOC Dermatologia, SAS Dermatologia Allergologica, Florence, Italy; h A.O.U. Citta della Salute e della Scienza di Torino, Turin, Italy; i San Gallicano Dermatological Institute - IRCCS, Rome, Italy; j U.O.C. Dermatologia A.S.P di Trapani, Trapani, Italy; k AO Ospedale Niguarda Ca’ Granda, Milan, Italy; l Novartis Farma SpA, Origgio, Italy; m ASST GOM Niguarda, Unit of Allergy and Immunology, Milan, Italy

**Keywords:** AWARE study, Italy, Chronic spontaneous urticaria, Socio-economic burden, Resource utilisation, CIndU, chronic inducible urticaria, CSU, chronic spontaneous urticaria, CU, chronic urticaria, CU-Q_2_oL, CU quality of life questionnaire, DLQI, dermatology life quality index, GCP, good clinical practices, H1-AH, H1-antihistamines, PRO, patient-reported outcomes, nsAH, non-sedating H1-AH, QoL, quality of life, sAH, sedating H1-AH, SD, standard deviation, UAS7, weekly urticaria activity score, WPAI-CU, work productivity and activity impairment questionnaire

## Abstract

**Introduction:**

In Italy, the real-world evidence on the extent of adherence to guidelines and the benefits of recommended therapeutic medications and their impact on the quality of life (QoL) of H_1_-antihistamines (H_1_-AH) refractory chronic urticaria (CU) patients is limited.

**Methods:**

AWARE (A World-wide Antihistamine-Refractory chronic urticaria patient Evaluation) was a global prospective, non-interventional study of CU in real-world setting which included patients aged ≥18 years with a medically confirmed diagnosed of CU present for more than 2 months. In this study, the disease characteristics, pharmacological treatments and patient-reported outcomes (PROs) are reported.

**Results:**

In total, 159 patients from 24 study centres in Italy completed the study. At baseline, 221 (89.5%) and 8 (3.2%) patients had chronic spontaneous urticaria (CSU) and chronic inducible urticaria (CIndU), respectively, while 18 (7.3%) patients had concomitant CSU and CIndU. For CSU patients, mean dermatology life quality index and CU quality of life questionnaire scores reduced to 3.0 ± 4.9 and 14.6 ± 18.6 at Month 24 from baseline scores of 7.5 ± 6.6 and 33.2 ± 19.5, respectively, indicating an improvement in QoL. This was reflected in their work-life as work productivity impairment reduced considerably after 2 years. Only 71.9% CSU patients had a prior treatment, while during the study, 96.8% of the patients were treated with a medication. At baseline, only 52.9% CSU patients reported nonsedating H_1_-antihistamines as first-line of treatment in prior medication, this increased to 89.6% during current medication.

**Conclusion:**

This study shows that CSU has a considerable socio-economic burden and an improvement in QoL can be achieved in CSU patients if an appropriate therapeutic path is followed.

## Introduction

Chronic urticaria (CU) is a common skin disorder that is associated with hives (wheals) and/or angioedema (swelling on the skin) for 6 weeks or more.^1^, ^2^, ^3^ CU can be generally classified into chronic spontaneous urticaria (CSU) and chronic inducible urticaria (CIndU). While CSU includes the spontaneous appearance of hives (wheals), CIndU can be triggered by external or any unknown inducing factors.^2^^,^^4^

The incidence of CU is higher in the age group of 20–40 years, although all age groups can be affected by the disease.^2^ Incidence among women is twice as high as men.^2^^,^^5^ CU has a significant negative impact on the quality of life (QoL) of patients. When compared to other skin diseases, the impact of CU is substantial due to associated symptoms that include itching, prominent red wheals, swellings in the deeper layers of the skin, and angioedema.^6^ All this triggers a loss in work productivity, lack of sleep, high levels of anxiety, and psychological distress.^2^^,^^7^

Here we report the socio-economic burden and resource utilisation for CU patients in the Italian subpopulation and as part of a larger global non-interventional study: A World-wide Antihistamine-Refractory chronic urticaria patient Evaluation (AWARE).^8^ Existing literature reports the CU prevalence in a community setting ranges between 0.5–3%.^9^, ^10^, ^11^ Annual prevalence of CSU in Italy was reported as 0.38% in year 2013 and is still on the rise.^9^

The international treatment guidelines recommend a step-by-step treatment approach for improving symptom control and for reducing the disease burden among CU patients. The first-line therapy includes approved doses of second-generation non-sedating H_1_-antihistamines (nsAH); however, nearly 50% of patients do not respond adequately and fail to achieve sufficient symptom control.^12^ In such cases, up-dosing (up to 4-fold of the recommended dose) is suggested as second-line therapy.^1^ In Italy, up-dosing of nsAH up to 4-fold is controversial and can cause serious problems for the allergist and dermatologist as the main side effects of AH are cardiotoxic effects and increased risk of QT prolongation.^13^^,^^14^ At the time of this study, the 2014 EAACI/GA^2^LEN/EDF/WAO guidelines recommended third-line therapy with omalizumab/montelukast or cyclosporine A if second-line therapy fails.^15^ The current 2017 EAACI/GA^2^LEN/EDF/WAO guidelines recommend adding omalizumab as an add-on therapy in patients unresponsive to high doses of nsAH.^1^ If the symptoms remain inadequately controlled with omalizumab after 6 months (or earlier if the symptoms are intolerable), add-on to nsAH therapy with cyclosporine A has been recommended as fourth-line treatment (off-label).^1^ Although an increase in dosage is especially effective in CIndU, a major problem with treatments in general with CU is angioedema that can occur concurrently with urticaria in up to 40% of the cases or even alone in up to 10%–20% of cases.^16^^,^^17^

The majority of previously published research on H_1_-refractory CU has samples drawn from specialised urticaria centres or small geographic areas.^6^^,^^10^ Minimal attention is given to assessment of disease burden in daily medical practice because tools like disease specific Chronic Urticaria Quality of Life Questionnaire (CU-Q_2_oL) are either not widely known or are perceived to be time consuming.^18^

Finally, there is limited real-world evidence as in the Italian subpopulation, on the extent of adherence to guidelines, the benefits of recommended therapeutic medications and their impact on the QoL of H_1_-AH refractory CU patients. In order to bridge this gap, the Italian subpopulation included patients who were refractory to the first-line therapy and recorded therapeutic effects in terms of symptom control and their influence in terms of improving the QoL at both specialised centres and private office-based practices.

## Methods

### Patients and study design

This report exclusively covers the results from the AWARE study in Italy. The AWARE study was a multicentre, prospective observational study conducted in 12 European countries. This study was designed to evaluate disease burden, current medication schedule, and use of clinical resources under conditions of daily living, with the following inclusion criteria: Patient consent to participate in the study; age ≥18 years; a clinical diagnosis of CU for more than 2 months and in patients with H_1_-AH refractory CU as a function of the administered therapy. Enrolled patients were followed-up for a period of 2 years. Exclusion criteria were: Absence of informed consent; patients' inability to attend regular follow-up visits, and simultaneous participation in any other CU study. The study was reviewed and approved by the Italian national and local ethics committees. The study was conducted in accordance with the Declaration of Helsinki (World Medical Association, 2008) and Good Clinical Practice (GCP) and in compliance with all local, and regional requirements, and written informed consent was obtained from each participant.

### Study outcomes

The primary objective was the correlation of patient-reported outcomes (PROs) with the treatment options used in patients with nsAH refractory CU. Treatment outcomes in terms of symptom control and QoL improvement were recorded for a period of 2 years. The co-primary endpoints were the courses of mean PROs measuring QoL: Dermatology Life Quality Index (DLQI), and disease activity: 7-day Urticaria Activity Score (UAS7) during the study. Secondary endpoints included: 1) Number of patients with CSU, CIndU, or both; 2) History of previous and current medications; 3) Number of visits of patients in different treatment groups; 4) Course of mean Chronic Urticaria QoL Questionnaire (CU-Q_2_oL), Work Productivity and Activity Impairment Questionnaire (WPAI), UAS7, and CIndU Score over the study; and 5) Frequencies of patients with respect to DLQI categories, and of utilisation of medical facilities by category; of patients with angioedema and wheals, their age at primary diagnosis and duration of the disease in years.

### Assessments

**Fig. 1:**
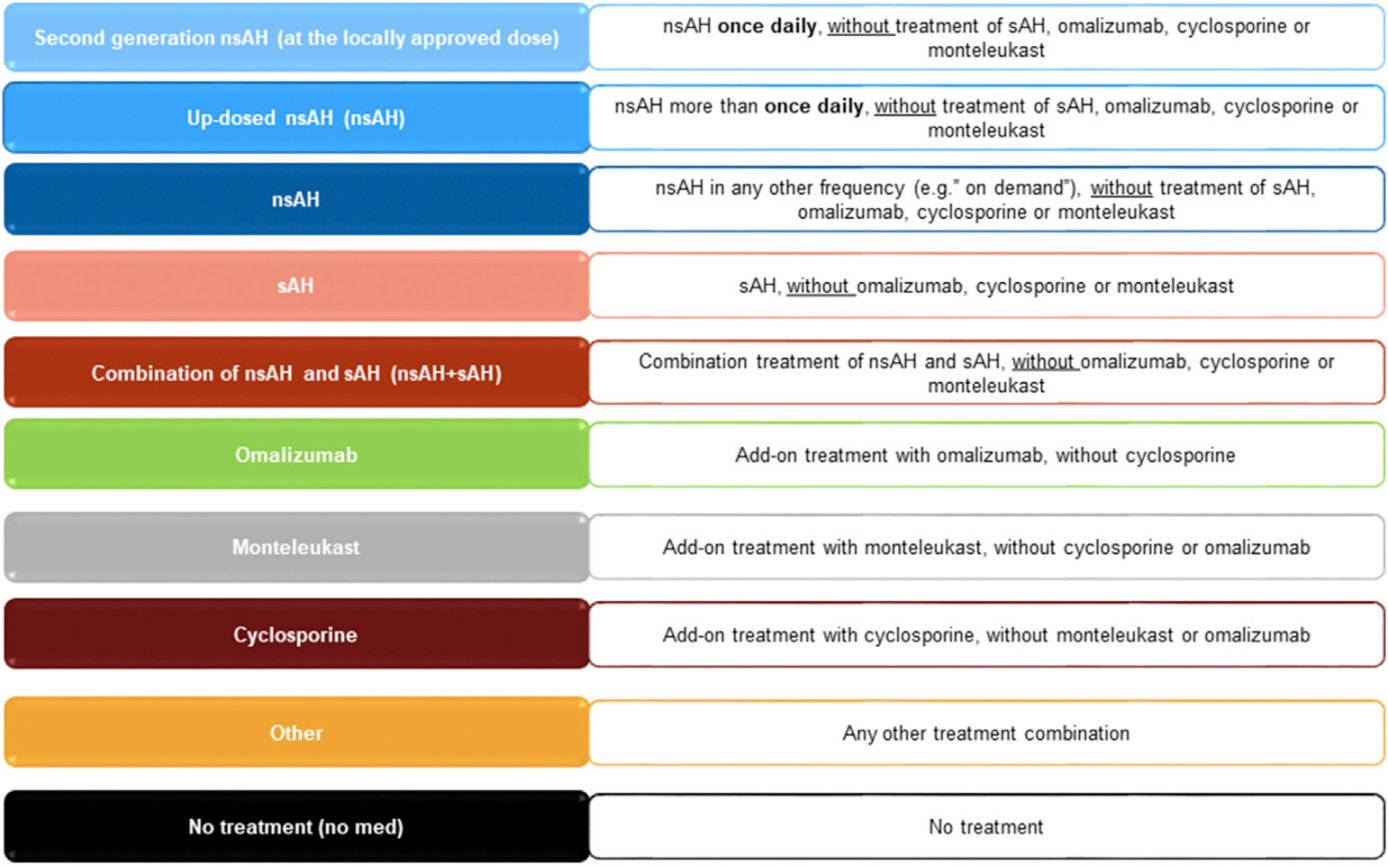
Predefined observed treatment groups

Patients were observed for a period of 2 years, with 8 visits at quarterly intervals following the visit at baseline. The various treatment groups examined are shown in Fig. 1. PRO measures used to assess disease activity, impact and control of the disease during this period included the UAS7,^19^, ^20^, ^21^, ^22^ DLQI,^23^^,^^24^ CU-Q_2_oL,^15^^,^^25^ and WPAI.^26^ Data were analysed and stratified according to the diagnostic groups: CSU, CIndU and both diagnoses (CSU+CIndU).

### Statistical methods

Only descriptive analyses were performed. Quantitative data including age were analysed by the statistical parameters valid N, missing N, mean, and standard deviation (SD). Qualitative data such as gender were presented by means of absolute and relative frequency distributions. The calculation of percentages was based on valid data per parameter and after excluding patients with missing values. Analyses of conditional endpoints (eg, duration of wheals longer than 24 h) were based on the number of subjects fulfilling the respective condition (eg, patients with wheals). All of the summary scores and respective sub-scores were presented using methods for quantitative variables. In addition to metric analysis, DLQI and the UAS7 were analysed categorically using the following thresholds: DLQI: 0–1, 2–5, 6–10, 11–20, 21–30 & UAS7: ≤6, >6.

## Results

### Patient disposition

Data from a total of 249 patients from 24 study centres (1–30 patients per study centre) were collected. Two patients (0.8%) were excluded from the analysis for not meeting the inclusion/exclusion criteria.

In total, 159 patients (64.4%) completed the study as scheduled. The most frequent reason for discontinuation for the remaining 88 patients was lost to follow-up (n = 37, 42.0% of patients with discontinuation), withdrawal of informed consent (n = 34, 38.6%), and spontaneous remission of CU/angioedema (n = 17, 19.3%).

### Demographics

Most of the patients (70.4%) enrolled in the study were females (Table 1). Of the 247 CU patients, 221 (89.5%) were diagnosed with CSU, 8 (3.2%) patients had CIndU, while 18 (7.3%) patients had concomitant CSU and CIndU. The most frequently reported diagnoses were CSU without angioedema in 121 (49.0%) patients and CSU with angioedema in 120 (48.6%) patients. Each of the other diagnostic categories (delayed pressure urticaria, urticaria factitia, cholinergic urticaria, cold urticaria, angioedema without wheals, contact urticaria, heat urticaria, light urticaria, and aquagenic urticaria) was observed in less than 3% of the patients. Any single diagnosis recorded for each patient was combined with one of three diagnostic groups: CSU, CIndU and both (CSU+CIndU). Most (157, 71.0%) of the CSU patients were women. The mean age (±SD) of CSU patients was 48.5 (±15.8) years, and at the baseline, the average duration of CSU was 5.0 (±7.1) years.

**Table 1:** Baseline demographics for the overall population and stratified by diagnostic category

Characteristic	CSU n = 221	CIndU n = 8	CSU + CIndU n = 18	Total N = 247
Age in years, mean ± SD	48.5 ± 15.8	42.4 ± 15.7	50.2 ± 13.9	48.4 ± 15.6
Female, n (%)	157 (71.0)	5 (62.5)	12 (66.7)	174 (70.4)
Duration of disease in years, mean ± SD	5.0 ± 7.1	3.9 ± 7.1	7.5 ± 8.4	5.1 ± 7.2
Family related history of urticaria, n (%)	18 (8.1)	0 (0.0)	5 (27.8)	23 (9.3)
BMI, n (mean) kg/m^2^	220 (25.8)	8 (25.3)	18 (25.9)	246 (25.8)
Current wheals or wheals in the last 6 months, n (%)	202 (92.7)	8 (100.0)	18 (100.0)	228 (93.4)
UAS7, mean ± SD	13.4 ± 13.4	0	42.0	16.3 ± 15.6
DLQI score, mean ± SD	7.5 ± 6.6	6.9 ± 8.2	8.6 ± 7.6	7.5 ± 6.7
DLQI categorical score, n (%)				
0–1: No effect at all	42 (19.1)	1 (12.5)	3 (16.7)	46 (18.7)
2–5: Little effect	69 (31.4)	5 (62.5)	5 (27.8)	79 (32.1)
6–10: Moderate effect	49 (22.3)	1 (12.5)	5 (27.8)	55 (22.4)
11–20: Large effect	50 (22.7)	0	3 (16.7)	53 (21.5)
21–30: Extremely large effect	10 (4.5)	1 (12.5)	2 (11.1)	13 (5.3)
Healthcare utilisation prior to baseline				
ER visits, mean ± SD (n [%])	2.9 ± 4.4 (86 [44.6])	3.0 (1[20.0])	5.1 ± 8.0 (8[44.4])	3.1 ± 4.8 (95 [44.0])
Hospitalisation visits, mean ± SD (n [%])	1.5 ± 0.9 (44 [22.8])	0	2.3 ± 1.9 (4 [22.2])	1.5 ± 1.0 (48 [22.2])
General practitioner visits, mean ± SD (n [%])	7.3 ± 9.9 (94 [48.7])	2.5 ± 0.7 (3 [60])	8.9 ± 6.6 (8 [44])	7.3 ± 9.6 (105 [48.6])
Allergologist/dermatologist visits, mean ± SD (n [%])	5.2 ± 6.6 (118 [61.1])	3.0 ± 1.4 (2 [40])	5.6 ± 6.7 (14 [77.8])	5.2 ± 6.5 (134 [62])
Specialist urticaria centre visits, mean ± SD (n [%])	5.4 ± 7.8 (84 [43.5])	6.7 ± 7.4 (3 [60])	24.0 ± 34.5 (7 [38.9])	6.6 ± 11.9 (94 [43.5])
Frequent co-morbidities at baseline, n (%)				
Hypertension	51 (23.1)	1 (12.5)	1 (5.6)	53 (21.5)
Allergic rhinitis	39 (17.6)	3 (37.5)	4 (22.2)	46 (18.6)
Anxiety disorder	29 (13.1)	0	2 (11.1)	31 (12.6)
Hashimoto's thyroiditis	26 (11.8)	0	2 (11.1)	28 (11.3)

BMI, body mass index; CIndU, chronic inducible urticaria; CSU, chronic spontaneous urticaria; DLQI, dermatology life quality index; ER, emergency room; HCU, healthcare utilisation; SD, standard deviation; UAS7, urticaria activity score over 7 days

### Patient-reported outcomes

#### Occurrence of itchy wheals and angioedema at baseline and during the study

**Fig. 2:**
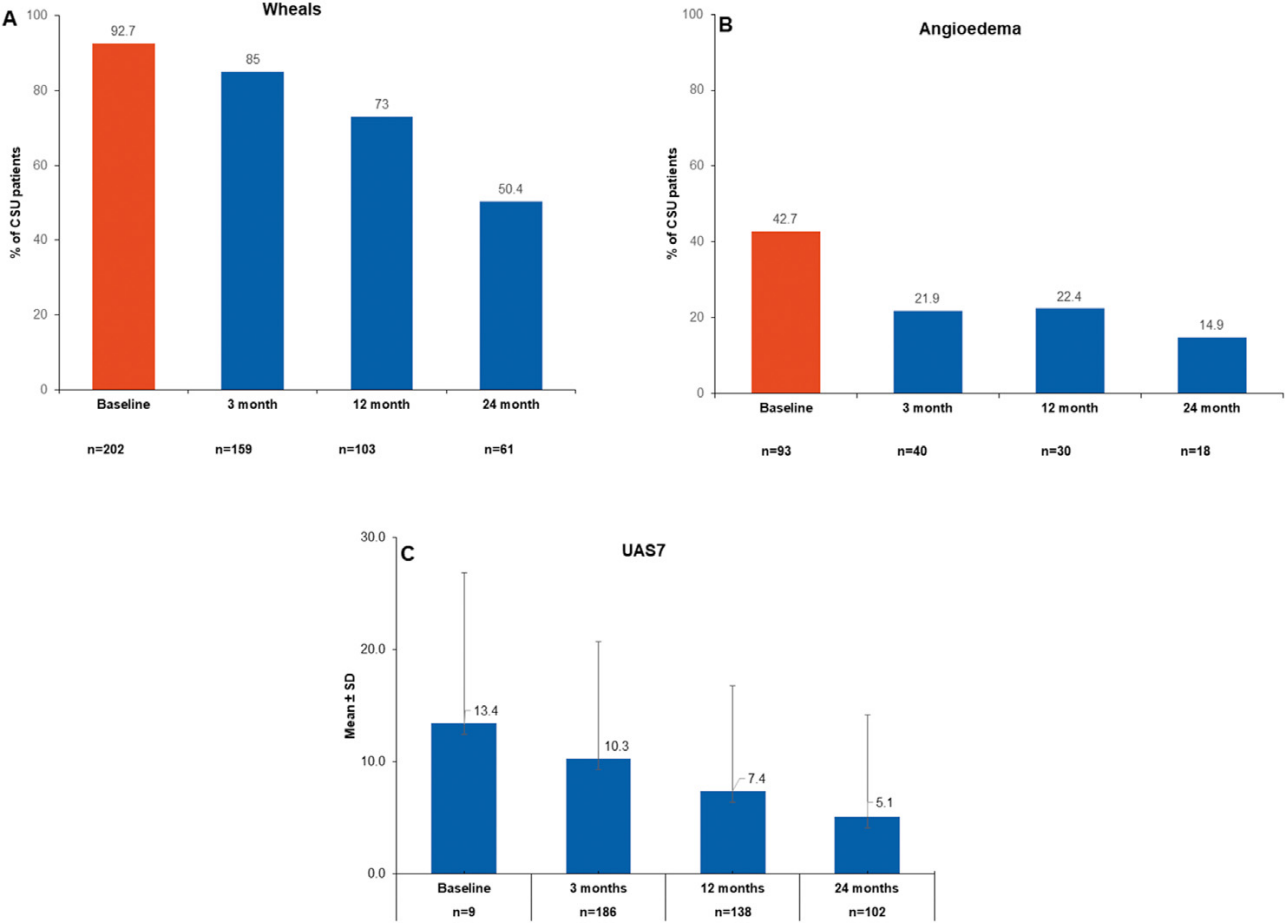
Prevalence of Wheals (2A) Angioedema (2B)UAS7 (2C) in patients with CSU over time. The number of subjects with available data at each visit varied because of the registry nature of AWARE.CSU, chronic spontaneous urticaria; n, number of patients; SD, standard deviation; UAS7; 7-day Urticaria activity score

At baseline, wheals (current or during last 6 months) were reported in 202 (92.7%) patients from a total of 221 patients diagnosed with CSU. Wheals for more than 24 h were reported in 49 (24.9%) CSU patients without a histological diagnosis of vasculitis. At Months 3, 12, and 24, 159 (85%), 103 (73%), and 61 (50.4%) patients with CSU reported wheals (Fig. 2A), respectively. At baseline, angioedema (in relation to urticaria) were reported in 93 (42.7%) patients with CSU and concomitant angioedema with itchy wheals appeared in 80 (86.0%) cases for an average of 20 h duration. While 8 (8.6%) patients reported angioedema that was triggered by a medical treatment, 3 (3.3%) patients reported treatment with angiotensin converting enzyme (ACE) inhibitors during the last 12 months before enrollment. At Months 3, 12, and 24, 40 (21.9%), 30 (22.4%), and 18 (14.9%) of patients with CSU reported angioedema (Fig. 2B), respectively.

#### UAS7 (7-day urticaria activity score)

At baseline, the UAS7 scores were available only for 9 patients. Mean UAS7 (±SD) for CSU patients declined over the study period from 13.4 (±13.4) at baseline to 5.1 (±9.1), N = 102 at Months 24 (Fig. 2C). For all treatment groups, the frequency of patients with high disease activity (UAS7 total score >6) decreased over the course of the study. A high proportion of patients treated with omalizumab were associated with urticaria that was well controlled during the subsequent visits, ranging from 10 (66.7%) patients at Month 3–13 (59.1%) patients at Month 12–14 (73.7%) patients at Month 24. However, after 2 years of observation, and depending on the treatment groups (on demand nsAH, n = 7; sAH, n = 2), 22.6%–50.0% of patients showed poor symptom control.

### Quality of life impairment in the 2-year observational period

#### Dermatology life quality index (DLQI)

**Fig. 3:**
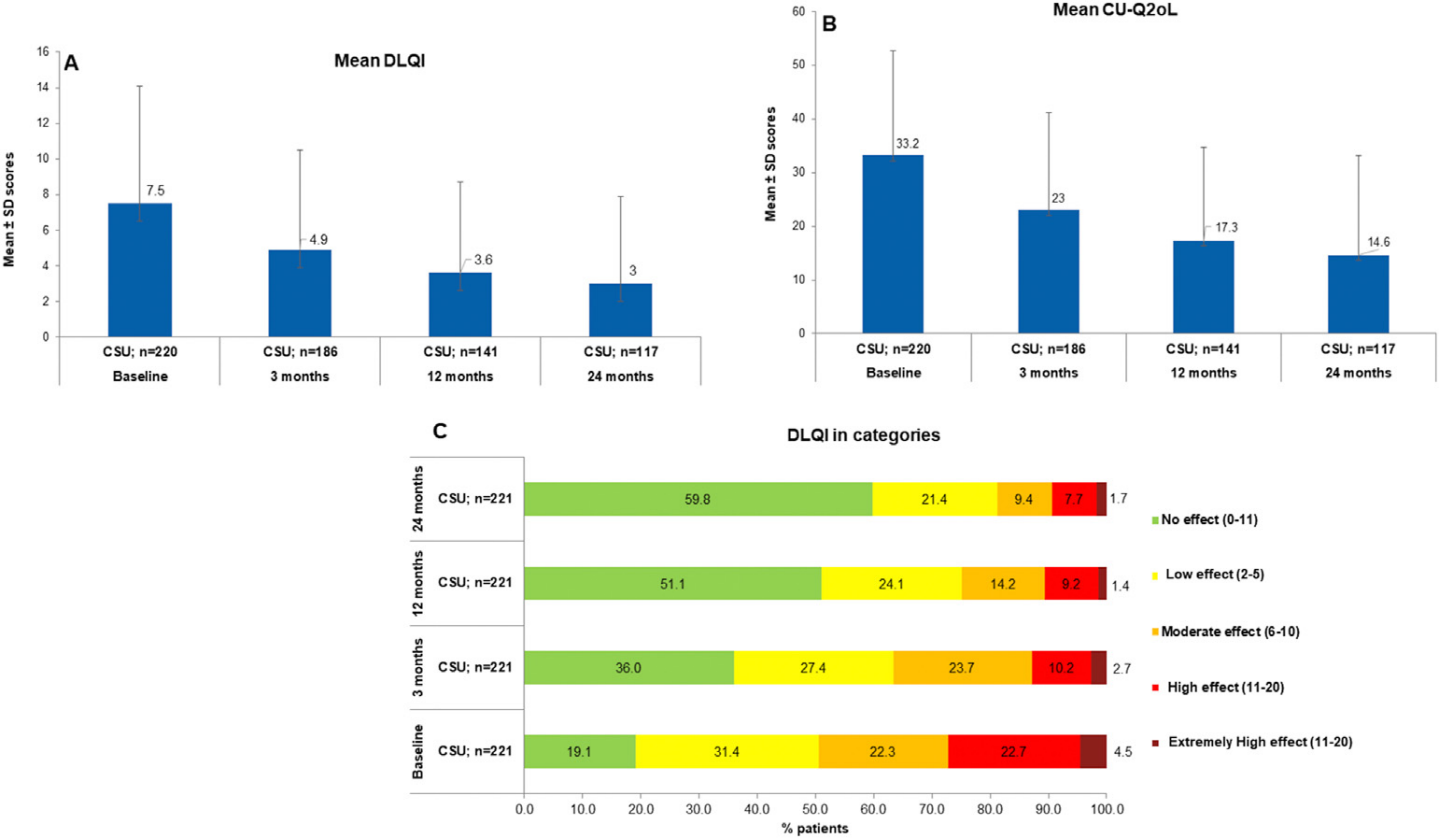
QoL of patients with CSU measured by DLQI (3A)CU-Q_2_oL (3B) DLQI (3C) in categories. The number of subjects with available data at each visit varied because of the registry nature of AWARE. CU-Q_2_oL, chronic urticaria quality of life questionnaire; DLQI, dermatology life quality index; n, number of patients; SD, standard deviation

The DLQI score decreased over the study period indicating an improvement in QoL. In the CSU population, the mean DLQI (±SD) declined from 7.5 (±6.6), N = 220 at baseline to 4.9 (±5.6), N = 186 at Month 3, 3.6 (±5.1), N = 141 at Month 12, and 3.0 (±4.9), N = 117 at Month 24 (Fig. 3A). Categorical DLQI scores at baseline showed that 109 (49.3%) patients with CSU experienced moderate to extremely high impact on their QoL (ie, DLQI total score ≥6) and merely 19.1% of CSU patients reported no effect at all on their QoL. This QoL improved to 67 (36%) at Month 3, and 72 (51.1%) at Month 12. At the end of the study, 70 (59.8%) of all CSU patients had no impact on QoL (Fig. 3C). However, after the 2-year observational period, 22 (18.8%) patients still reported to have at least a moderate or worse impact on their QoL due to CSU.

#### Chronic urticaria quality of life questionnaire (CU-Q_2_oL)

The mean CU-Q_2_oL (±SD) for CSU patients decreased from 33.2 (±19.5), N = 220 at baseline to 23.0 (±18.2), N = 186 at Month 3; 17.3 (±17.4), N = 141 at Month 12 and subsequently to 14.6 (±18.6), N = 117 at Month 24, indicating a clear improvement of the patients’ QoL during the course of the study (Fig. 3B).

### Healthcare resource utilisation and prior treatment

#### Utilisation of medical and clinical resources

Prior to baseline, 86 (44.6%) of 221 CSU patients visited an emergency physician or an emergency room and 44 (22.8%) were hospitalised at least once. At Month 3, 12, and 24, CSU patients either visited an emergency physician or an emergency room (1 [2.6%], 3 [15.0%], and 2 [14.3%]) or were hospitalised at least once (1 [2.6%], 1 [5.0%], and 0 [0.0%]), respectively. At baseline, 94 (48.7%) CSU patients visited a general practitioner or family physician, 84 (43.5%) CSU patients visited a specialised urticaria centre, and 118 (61.1%) CSU patients further visited a dermatologist or allergist. At Month 3, 12, and 24, 6 (15.4%), 2 (10.0%), and 2 (14.3%) CSU patients visited a general practitioner; 15 (38.5%), 7 (35.0%), and 2 (14.3%) CSU patients visited a specialised urticaria center and 12 (30.8%), 2 (10.0%), and 7 (50.0%) CSU patients visited a dermatologist or allergist, respectively. Additionally, only 25 (11.3%) CSU patients had reported sick leave due to urticaria (since the time of diagnosis) at baseline with an average duration of 4.8 weeks. At Month 3, 12, and 24, 6 (3.2%), 2 (1.4%), and 1 (0.8%) patients reported sick due to urticaria, respectively.

#### Prior medication and treatment groups

**Fig. 4:**
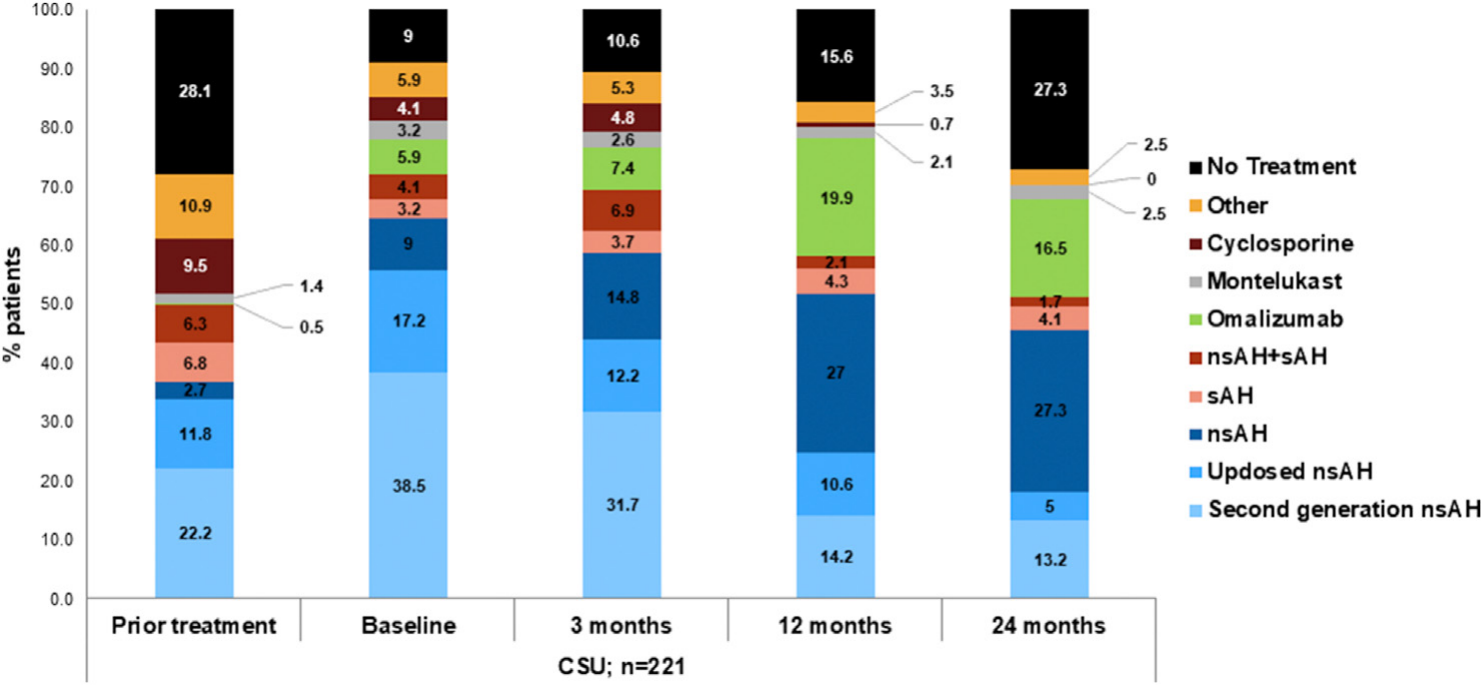
Treatment groups over the 2-year study period in patients diagnosed with CSU. *CSU,* chronic spontaneous urticaria; n, number of patients; nsAH, non-sedating antihistamines; sAH, sedating antihistamines

A total of 71.9% of CSU patients received a prior medication for urticaria. Prior to baseline, the most frequent medication prescribed was nsAH with 52.9% of the CSU patients followed by corticosteroids and sAH prescribed in 37.1% and 16.7% of the CSU patients respectively. Before the baseline visit, 28.1% of all the patients had no treatment for CSU, while only 35.9% of the patients were on guideline recommended treatments (first-line approved nsAH in 22.2% patients, second-line up-dosed nsAH in 11.8% patients, third-line leukotriene inhibitor montelukast in 1.4% patients, and fourth-line anti-IgE antibody omalizumab in just 0.5% patients) (Table 2). Treatments not recommended by guidelines were prescribed in 24% patients with CSU (combination of sAH and nsAH in 6.3% patients, sAH in 6.8% patients, and other non-recommended treatment in 10.9% patients) and more than a quarter of the patients (28.1%) did not receive any treatment for CSU prior to baseline (Table 2). A reduction in use of cyclosporine treatment was recorded during the observation period, from 9.5% before enrollment to 0 at month 24. A reduction in use of sAH, combination of sAH and nsAH, and other non-recommended treatment was noticed at Month 24 (Fig. 4). The most noticeable changes in medication intake during the study period were observed for nsAH treatment with 38.5% patients at baseline to 13.2% patients at Month 24, on demand nsAH treatment with 9.0% patients at baseline to 27.3% patients at Month 24, and up-dosed nsAH treatment from 17.2% patients at baseline to 5.0% patients at Month 24. Proportions of patients with omalizumab treatment increased during the study from 5.9% patients at baseline to 16.5% patients at Month 24 (Fig. 4).

**Table 2:** Prior medication for urticaria at baseline stratified by diagnostic category

Treatment	CSU n = 221; n (%)	CIndU n = 8; n (%)	CSU+CIndU n = 18; n (%)	Total N = 247; n (%)
**Any treatment**	159 (71.9)	5 (62.5)	11 (61.1)	175 (70.9)
nsAH	117 (52.9)	3 (37.5)	8 (44.4)	128 (51.8)
Corticosteroid	82 (37.1)	0 (0)	3 (16.7)	85 (34.4)
sAH	37 (16.7)	0 (0.0)	4 (22.2)	41 (16.6)
Cyclosporine	25 (11.3)	0 (0.0)	2 (11.1)	27 (10.9)
Montelukast	6 (2.7)	1 (12.5)	1 (5.6)	8 (3.2)
Omalizumab	2 (0.9)	1 (12.5)	1 (5.6)	4 (1.6)
**Treatment groups for urticaria**				
No treatment	62 (28.1)	3 (37.5)	7 (38.9)	72 (29.1)
nsAH approved	49 (22.2)	0 (0.0)	1 (5.6)	50 (20.2)
Up-dosed nsAH	26 (11.8)	2 (25.0)	2 (11.1)	30 (12.1)
Other non-recommended treatment^a^	24 (10.9)	1 (12.5)	0 (0.0)	25 (10.1)
Cyclosporine	21 (9.5)	0 (0.0)	2 (11.1)	23 (9.3)
Combination of nsAH and sAH	14 (6.3)	0 (0.0)	3 (16.7)	17 (6.9)
sAH	15 (6.8)	0 (0.0)	1 (5.6)	16 (6.5)
Omalizumab	1 (0.5)	1 (12.5)	1 (5.6)	3 (1.2)
Montelukast	3 (1.4)	1 (12.5)	1 (5.6)	5 (2.0)

Only medications applied by >1% of the patients are presented. CSU, chronic spontaneous urticaria; CIndU, chronic inducible urticaria; n, number of patients; N, Total number of patients; nsAH, Non-sedating H_1_-Antihistaminines; sAH, Sedating H_1_-Antihistaminines.

a Other non-recommended treatment refers to any other treatment combination

A total of 96.8% were treated with medication during the study period. A majority of the patients (89.6%) were treated with nsAH and 34.8% patients were treated with corticosteroid followed by omalizumab in 20.4% patients.

### WPAI and total activity impairment

**Fig. 5:**
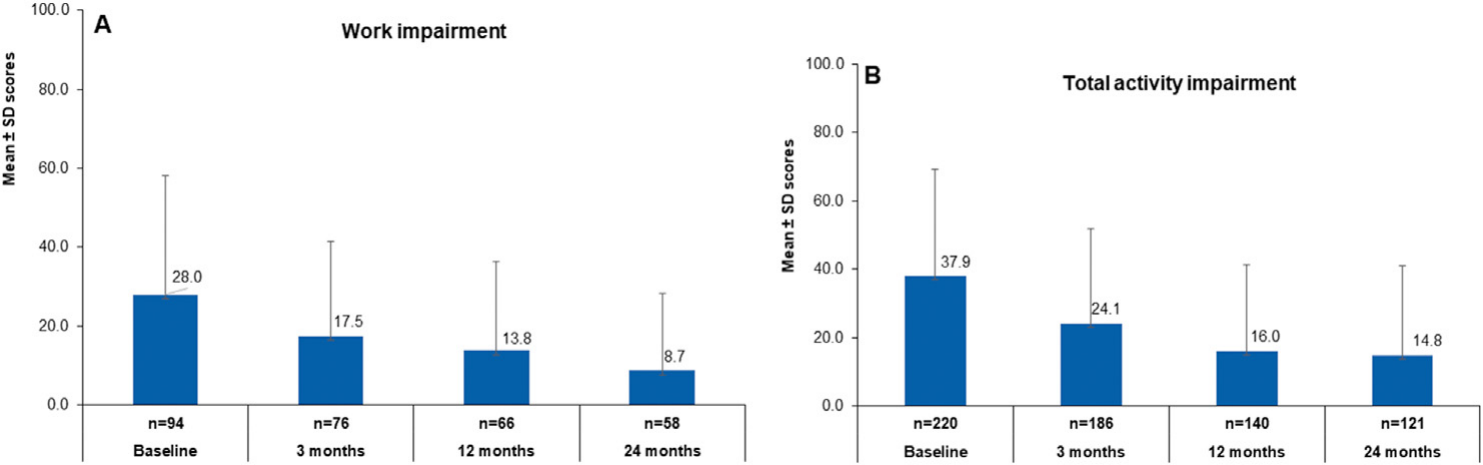
Percentage of A. Total work productivity impairment B. Total activity impairment. The number of subjects with available data at each visit varied because of the registry nature of AWARE. n, number of patients; SD, standard deviation

CSU patients showed considerable improvement in daily activity and work productivity measured by the WPAI. The mean percentage of work impairment (±SD) at baseline was 28.0% (±30.0), which reduced to 13.8% (±22.5) at Month 12. In the second year (Month 24), the percentage of total work productivity impairment further reduced to 8.7% (±19.5) (Fig. 5A). The mean percentage of total activity impairment (±SD) by CSU was 37.9% (±31.4) at baseline. This means that the patients’ ability to do regular daily activities was impaired by more than one-third on average during this time. This impairment was reduced during the first year of the study to 16.0% (±25.4). In the second year, total activity impairment further declined to 14.8% (±26.1) (Fig. 5B).

## Discussion

The present data represent the real-world burden of disease in CSU patients along with the treatment patterns in Italy. The demographics of enrolled patients largely resemble the published data from CSU clinical trials^27^, ^28^, ^29^ with 70.4% females, a mean age of 48 years, and a mean BMI of 25.8 kg/m^2^. The most frequent reason stated for early discontinuation of the study was “lost to follow-up” followed by “withdrawal of informed consent”, and spontaneous remission of CU. A large majority of the patients (89.5%) were diagnosed with CSU; concomitant CSU and CIndU was observed in 7.3% of patients while CIndU was observed in only 3.2% patients, which is in contrast to previous literatures that reported concomitant CSU and CIndU in as high as 24% of patients in other European regions.^30^^,^^31^ Anxiety disorders were relatively high in the Italian AWARE population (13.1%) compared to other cohorts of the AWARE study.

Data for the Italian cohort of the AWARE study were collected during the period when the treatment guidelines for urticaria were defined by the 2014 EAACI/GA^2^LEN/EDF/WAO urticaria guideline (relevant at the time of the AWARE study). The first-line therapy included approved doses of second-generation nsAH, followed by dose escalation of second-generation nsAH up to 4 times the approved dose as second-line therapy. Third-line add-on therapy included cyclosporine and thereafter escalation to add-on to nsAH omalizumab/montelukast when patients displayed insufficient symptom control. These guidelines discouraged long-term use of first-generation H1-AH/sAH or corticosteroids. An update of the EAACI/GA^2^LEN/EDF/WAO urticaria guideline in 2017 now recommends omalizumab as a standard third-line therapy before cyclosporine and a short course of glucocorticosteroids is permitted in case of severe exacerbation of CSU.^1^ In general, the patients were not receiving the treatment in accordance with the guidelines prior to the study. A large proportion of the Italian patient's population was undertreated for urticaria, which is similar to previous AWARE study reports for other regions.^30^^,^^31^

Prior to baseline, more than a quarter of the CSU patients did not receive treatment for urticaria, and nearly a quarter of patients received non-recommended treatments. Corticosteroid usage was reported in 37.1% of the CSU patients, which was higher than the German (15.8%) and Scandinavian (19%) cohorts of the AWARE study previously reported.^30^^,^^31^ Additionally, 16.7% of the CSU patients used sAH, which was also higher than the German (10%) and the Scandinavian (3.2%) cohorts.^30^^,^^31^ The overall use of omalizumab was moderate because omalizumab received approval from AIFA (Italian Health Authority) in August 2015 and was available in the Italian market in September 2015. The recommended treatment options of nsAH at approved and escalated doses were the most frequently prescribed medications, although the treatment regimens wildly varied. Omalizumab was the only approved treatment for CSU in case of treatment failure or treatment nonresponse to nsAH; it was prescribed in 20.4% of the CSU patients.

A total of 49.3% patients had moderate to extremely high impact on their QoL at baseline assessed by DLQI. There was substantial improvement in symptoms of CSU over the 2-year observational period as evidenced at all investigated endpoints. Although an improvement in the QoL was achieved following an appropriate therapeutic path and as a function of the administered therapy, a large proportion of CSU patients were still considered "uncontrolled" after a 2-year period and after being seen by a specialist.

Concomitant angioedema was reported in nearly half of the CSU patients and was present on average for 20 h within the preceding 6 months. Angioedema also appeared together with itchy wheals in the majority of cases (86.0%) and reported to be of moderate disease intensity. A reduction in concomitant angioedema and wheals was noticed over the 24 months of the observation period. Yet a considerable number of patients still had high disease burden after 24 months because wheals and angioedema were still present in 50.4% and 15% of patients, respectively. A reduction in these symptoms compares well with the improvements in urticaria activity score and QoL assessed throughout the observation period.

CSU patients demonstrated a high rate of medical resource utilisation at baseline, and in many cases, multiple dermatologists or allergists were consulted in addition to the general practitioner involvement. More than 20% of the patients experienced hospitalisation due to CSU at baseline. Scarce information was available regarding the number of patients utilising medical and clinical resources at Month 12 and Month 24; thus, a strong conclusion was not drawn from the data. At baseline, 11.3% of all patients reported at least 1 sick leave due to CSU since the onset of disease which was considerably lower than that observed in the general AWARE population. In comparison to Germany and Scandinavia, 27% of patients reported having sick leaves due to urticaria with an average duration of 9 weeks, and 25.9% of patients reported sick leaves due to urticaria with an average duration of 3.8 weeks per year, respectively. Absenteeism was high in patients with CU as indicated in previous studies of the literature.^3^ However, during the 2-year observation period, Italian patients rarely took any sick leave, probably to safeguard their job/employment.

Although the assessment of the real-world practice was the main limitation of the AWARE study, this is one of the first studies in Italy with a large number of CU patients followed up over a long period. There were no pre-defined or randomised group assessments of patients; instead, data describing the treatment strategy of a patient were assessed on an ongoing basis and could vary during the study. Inclusion criteria were fairly broad and no explicit exclusion criteria apart from anticipated difficulties in patient follow-up and simultaneous participation in any other clinical trial for urticaria were applied. Due to which, additional factors causing a selection bias cannot be completely ruled out. The data obtained with this study were assessed using descriptive statistics and were not used for comparative analysis.

In conclusion, the data included here show the disease burden and the treatment regimens of CSU patients in Italy. Untreated or sub-optimally treated CU patients have a high disease burden which contributes to higher economic burden and higher utilisation of healthcare resources. Adherence to the latest treatment guidelines for treatment and managing patients can improve the patient care and disease outcomes. The outcomes from this report may further contribute to the understanding of the disease burden and the treatment algorithm for CU practised in Italian patients.

## Ethics

The study was reviewed and approved by the Italian national and local ethics committees. The study was conducted in accordance with the Declaration of Helsinki (World Medical Association, 2008) and Good Clinical Practice (GCP) and in compliance with all local, and regional requirements, and written informed consent was obtained from each participant.

## Author contributions

OR, CC, and RC designed the study. RI, SS, MPF, GV, VV, CP, LR, NS contributed to the clinical and laboratory work for the study. RI and SS contributed to data collection. AN contributed to data analysis. RC, CC, and NC drafted the article and revised it critically for important intellectual content. RC, CC, and NC contributed to final approval of the version to be published. All authors contributed to drafting, revising and editing the article, gave final approval of the version to be published, and agree to be accountable for all aspects of the work.

OR and FS substantially contributed to conception and design of the analysis and interpretation of the data. All authors contributed to data collection, to drafting, revising and editing the article, gave the final approval of the version to be published, and agree to be accountable for all aspects of the work.

## Data availability

The datasets generated during and/or analysed during the current study are not publicly available. Novartis is committed to sharing with qualified external researchers access to patient-level data and supporting clinical documents from eligible studies. These requests are reviewed and approved the basis of scientific merit. All data provided is anonymized to respect the privacy of patients who have participated in the trial in line with applicable laws and regulations. The data may be requested from the corresponding author of the manuscript.

## Submission declaration

The work has not been published or submitted to another scientific journal and is not being considered for publication elsewhere. This submission represents original work and is approved by all authors.

## Funding

The study was supported by Novartis Pharma AG Acknowledgements: The authors would like to thank Bitumani Borah, DVM, MSc and Sumeet Sood, PhD (Novartis Health Care Pvt. Ltd., Hyderabad, India) for providing medical writing and editorial assistance.

## Declaration of competing interest

O Rossi: Alk-abello, Italchimici and GSK. E Iemoli: Served as a medical doctor in ASST FBF-Sacco Milano. Prof A Patrizi: Principal investigator for Abbvie, Eli Lilly, Leo, Novartis, Sanofi Genzyme Regeneron, Pfizer and speaker, advisory board member and/or consultant for Sanofi Genzyme Regeneron, Menarini, Abblie, Lilly, Pierre fabre, La roche Posay, Novartis, Leo, Almirall, Celgene. L Stingeni: Principal investigator in clinical trials sponsored by Novartis and has served on advisory board from Novartis. S Calvieri: Abiogen. M Gola: Sanofi e Beiersdorf. P Dapavo: Novartis, Abbvie, Celgene, Lilly spa, Leopharma, Sandoz, Janssen. L Zichichi: Principal Investigator in clinical trials sponsored by Novartis and has served on advisory board from Novartis. F Saccheri: Employee, Novartis. All other authors have noting to disclose.
